# Association between multidimensional prognostic index (MPI) and pre-operative delirium in older patients with hip fracture

**DOI:** 10.1038/s41598-022-20734-2

**Published:** 2022-10-08

**Authors:** Clarissa Musacchio, Carlo Custodero, Monica Razzano, Rita Raiteri, Andrea Delrio, Domenico Torriglia, Marco Stella, Matteo Puntoni, Carlo Sabbà, Antonella Barone, Alberto Pilotto

**Affiliations:** 1 Department of Geriatric Care, OrthoGeriatrics and Rehabilitation, Frailty Area, E.O. Galliera Hospital, Via Mura delle Cappuccine 16, 35121 Genoa, Italy; 2 Department of Interdisciplinary Medicine, Clinica Medica e Geriatria “Cesare Frugoni”, University of Bari Aldo Moro, Bari, Italy; 3 Orthopedic Unit, Department of Geriatric Care, OrthoGeriatrics and Rehabilitation, Frailty Area, E.O. Galliera Hospital, Genoa, Italy; 4 Clinical Trial Unit, Scientific Directorate, E.O. Galliera Hospital, Genoa, Italy; 5 Clinical and Epidemiological Research Unit, University Hospital of Parma, Parma, Italy

**Keywords:** Neurology, Risk factors, Bone

## Abstract

Pre-operative delirium may cause delay in surgical intervention in older patients hospitalized for hip fracture. Also it has been associated with higher risk of post-surgical complications and worst functional outcomes. Aim of this retrospective cohort study was to evaluate whether the multidimensional prognostic index (MPI) at hospital admission was associated with pre-operative delirium in older individuals with hip fracture who are deemed to require surgical intervention. Consecutive older patients (≥ 65 years) with hip fracture underwent a comprehensive geriatric assessment to calculate the MPI at hospital admission. According to previously established cut-offs, MPI was expressed in three grades, i.e. MPI-1 (low-risk), MPI-2 (moderate-risk) and MPI-3 (high risk of mortality). Pre-operative delirium was assessed using the four ‘A’s Test. Out of 244 older patients who underwent surgery for hip fracture, 104 subjects (43%) received a diagnosis of delirium. Overall, the incidence of delirium before surgery was significantly higher in patients with more severe MPI score at admission. Higher MPI grade (MPI-3) was independently associated with higher risk of pre-operative delirium (OR 2.45, CI 1.21–4.96). Therefore, the MPI at hospital admission might help in early identification of older patients with hip fracture at risk for pre-operative delirium.

## Introduction

Delirium is a syndrome of acute change in cognition and alertness, often associated with psychotic behavior^1^. According to current estimates, delirium, with an incidence ranging from 14 to 56%, is one of the commonest complication in hospitalized older adults^2^ and is even more frequent in subjects undergoing urgent surgery^3^. Hip fracture represents the first cause of urgent surgery among older adults with over 300,000 hospital admissions each year in the United States^4^. In these patients, delirium usually occurs within the first 24–48 h after surgery with a frequency ranging between 20 and 50%^5,6^. However, most of the patients suffering from delirium already show it pre-operatively^7^. This may cause surgical delays which on turn increases delirium risk^8–10^ . Pre-operative delirium is associated with worst prognosis compared to post-operative delirium including prolonged hospitalization, higher request of healthcare resources, loss of independence, institutionalization and death^11–13^.

Nevertheless, systematic assessment is not yet universally applied and delirium is often underdiagnosed^14^. Considering the relevant health impact of delirium^6^, and the growing number of older adults undergoing falls with hip fracture^4^, there is a need of instruments able to early identify subjects more at risk to develop delirium since the pre-operative phases. This could help to design strategies for earlier and more appropriate intervention. It is becoming clear that multidimensional impairment of older patients may influence the clinical outcome of acute diseases^15^. It is also evident that etiology of delirium is likely multifactorial and linked to many other geriatric syndromes (e.g. falls, functional decline, frailty, dementia)^7,9,13,16^. Recently, it has been shown that the multidimensional prognostic index (MPI), a predictive tool of mortality, based on a standardized comprehensive geriatric assessment (CGA), is an independent predictor of 6-month mortality in older patients with hip fracture^17^.

The aim of the present study was to evaluate the usefulness of the MPI for prompt detection of older patients admitted to the hospital for hip fracture surgery and who were at risk for pre-operative delirium.

## Methods

### Study population

**Figure 1:**
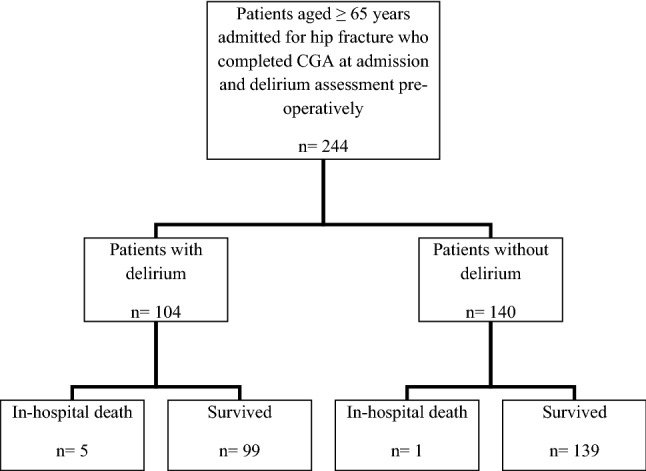
Retrospective cohort study flowchart.

We conducted a retrospective observational cohort study on consecutive patients aged 65 years and older admitted from January 2017 to December 2017 to the OrthoGeriatrics Unit of Galliera Hospital of Genoa, Genoa, Italy. Inclusion criteria were: (a) age ≥ 65 years, (b) diagnosis of hip fracture, (c) complete CGA at admission, (d) assessment of delirium during hospitalization, and (e) ability to provide an informed consent or availability of a proxy for informed consent. There were no specific exclusion criteria. Patients’ flowchart is presented in Fig. 1. Information on waiting time for surgery, type of anesthesia (i.e. general, spinal anesthesia alone or with peripheral nerve block, other types), presence of infections or anemia after surgery, and length of hospitalization were recorded.

Informed consent was obtained from all participants and/or their legal guardians. This study was conducted following the Strengthening the Reporting of Observational Studies in Epidemiology (STROBE) guidelines^18^ and adhered to the tenets of the World Medical Association’s Declaration of Helsinki.

### The multidimensional prognostic index

The multidimensional prognostic index (MPI) is a well validated tool measuring multidimensional frailty^15^. At baseline the MPI was calculated from the data derived from a standard CGA which included information on the following eight domains: functional status evaluated with the activity of daily living (ADL)^19^ and the instrumental ADL (IADL) scales^20^; cognitive status evaluated by the Short Portable Mental Status Questionnaire (SPMSQ)^21^; nutritional status evaluated by the Mini Nutritional Assessment-Short Form (MNA-SF)^22^; risk of developing pressure sores evaluated by the Exton Smith Scale (ESS)^23^; co-morbidity was examined using the Cumulative Illness Rating Scale (CIRS)^24^; number of drugs assumed by patients at admission and the co-habitation status (i.e. alone, in family or in institute) were also recorded. According to previous established cut-off, MPI was expressed in three grades, i.e. MPI-1 (low-risk risk MPI value ≤ 0.33), MPI-2 (moderate-risk MPI value between 0.34 and 0.66) and MPI-3 (high risk of mortality MPI value > 0.66)^25^.

### Diagnosis of delirium

Screening of delirium was made before surgery using the four ‘A’s Test (4AT). The 4AT is a composite test which assigns a score to the four following components: (a) alertness (0: fully alert or mild sleepiness for < 10 s after waking; 4: abnormal), (b) the Abbreviated Mental Test-4 for recall of age, date of birth, place (name of the hospital) and current year (0: no mistakes; 1: one mistake; 2: two or more mistakes or untestable), (c) attention assessed asking the patient to list months backwards starting from December (0: reciting ≥ 7 months correctly; 1: starts but recites < 7 months or refuses to start; 2: untestable), (d) acute change or fluctuating course in mental status within the last 2 weeks and persisting in the last 24 h (0: no; 4: yes). Summing the scores of the four components, we obtain a total score ranging from 0 to 12 with 0 indicating low probability to have delirium or severe cognitive impairment; 1–3: possible cognitive impairment and does not exclude the possibility of delirium; 4 or more suggesting possible delirium with or without cognitive impairment^26^. Diagnosis of delirium was met whether the suspect based on 4AT score was confirmed by criteria of the Diagnostic and Statistical Manual of Mental Disorders, Fifth Edition (DSM-V)^1^.

### Statistical analysis

Descriptive statistics were used for continuous factors and expressed as mean and standard deviation or median and interquartile range (IQR). In case of categorical factors, absolute and relative frequencies (%) were reported. Fisher's exact test was used to compare categorical factors. Normality of data was tested graphically (histograms and quantile–quantile plot) and through formal test (Shapiro–Wilk W test); independent sample t-test (in case of normally distributed data) or Mann–Whitney test (in case of not normally distributed data) were used for comparison of continuous variables between subjects with and without diagnosis of delirium. Logistic regression modelling was adopted to calculate odds ratios (ORs) and Wald test to estimate and test the association between the diagnosis of pre-operative delirium and pre-operative (gender, age, social support network, number of drugs, MPI score, waiting time for surgery), intra-operative (type of anesthesia) and post-operative factors (infections, anemia). All analyses were conducted using Stata (version 14.2, StataCorp, College Station, TX, USA) software. Two-tailed probabilities were reported and a *p* value of 0.05 was used to define nominal statistical significance.

### Ethical approval and informed consent

The study received formal ethical approval by the Ethical Committee of Regione Liguria, Italy. All participants gave written informed consent.

## Results

Overall, 244 older adults with a diagnosis of hip fracture were eligible for the study. Patients were mainly female (84.2%) with a mean age of 85 (6.9) years, ranging from 65 to 102 years old. Before surgery, delirium occurs in 104 subjects (43%). Table 1 shows the baseline clinical and functional characteristics of patients according to diagnosis of delirium. Patients with delirium were significantly older (87 ± 6.2 vs. 83 ± 6.8, *p* < 0.001), with a similar proportion of females (85.6% vs. 83.2%, *p* = ns) compared to patients without delirium. Length of stay was about 3 days longer in patients with delirium [11 (IQR, 8–15) vs. 8 (IQR, 6–11), *p* = 0.04], as well as post-operative complications like anemia (80.8% vs. 60.1%, *p* < 0.001) and infections (58.7% vs. 41.4%, *p* < 0.001) were significantly more frequent among subjects with pre-operative delirium. Differences were also detected in the type of anesthesia adopted. Death during hospital stay occurred in 5 (4.8%) patients with delirium and 1 (0.7%) without delirium (Fisher exact *p* = 0.052).

**Table 1:** Characteristics of patients by presence of pre-operative delirium.

	Delirium (n = 104)	No delirium (n = 140)	Overall (n = 244)	*p* Value*
**Age**, *years*, mean (SD)	87 (6.2)	83 (6.8)	85 (6.9)	< 0.001
**Sex, n (%)**				
Male	15 (14.4)	24 (17.1)	39 (16.0)	0.6
Female	89 (85.6)	116 (82.8)	205 (84.0)	
**Length of stay**, *days,* median (IQR)	11 (8–15)	8 (6–11)	9 (7–13)	< 0.001
**Time to surgery**, *days*, median (IQR)	3.5 (2–5)	3 (2–4)	3 (2–4)	0.01
**Type of anesthesia, n (%)**				
Spinal	51 (49.0)	56 (40.0)	107 (43.9)	0.04
Spinal + nerve block	46 (44.2)	61 (43.6)	107 (43.9)	
General	3 (2.9)	3 (2.1)	6 (2.5)	
Other	4 (3.9)	20 (14.3)	24 (9.8)	
**Post-operative anemia, n (%)**	84 (80.8)	85 (60.7)	169 (69.3)	0.001
**Post-operative infection, n (%)**	61 (58.7)	48 (34.3)	109 (44.7)	< 0.001

*IQR* interquartile range, *SD* standard deviation.

*p Value for *t* test (age), Mann–Whitney test (length of stay and time to surgery) or Fisher exact test.

Table 2 shows MPI score and its subdomains according to diagnosis of delirium. Patients who developed pre-operative delirium, had higher MPI score at admission compared to those without delirium [median 0.75 (IQR, 0.63–0.81) vs. 0.63 (IQR, 0.56–0.75), *p* < 0.001]. No MPI-1 class patients were found in our cohort. Patients with delirium were more likely to be in the MPI-3 class (highest mortality risk) at admission compared to patients without delirium (73% vs. 48%, *p* < 0.001). Considering MPI domains, patients with delirium had lower IADL [median 1 (IQR, 0–2) vs. 2 (IQR, 1–4.5), *p* < 0.001], SPMSQ [4 (IQR, 0–8) vs. 9 (IQR, 6–10), *p* < 0.001] and MNA-SF [8 (IQR, 5–10) vs. 9 (IQR, 7–11), *p* = 0.004] than those without delirium; but no significant difference was found in ADL and CIRS score between the two patients’ groups. The number of drugs assumed by patients was similar in the two groups. As regards social support network, 110 (45.1%) lived in the family, 33 (13.5%) were institutionalized and 101 (41.4%) lived alone, without any difference between subjects who presented or not delirium.

**Table 2:** MPI score and other subdomains according to diagnosis of delirium.

	Delirium (n = 104)	No delirium (n = 140)	*p* Value*
**MPI classes, n (%)**			
2	28 (26.9)	73 (52.1)	< 0.001
3	76 (73.1)	67 (47.9)	
MPI, median (IQR)	0.75 (0.63–0.81)	0.63 (0.56–0.75)	< 0.001
ADL, median (IQR)	0 (0–1)	1 (0–1)	0.1
IADL, median (IQR)	1 (0–2)	2 (1–4.5)	< 0.001
CIRS, median (IQR)	4 (2.5–5)	4 (2–5)	0.8
SPMSQ, median (IQR)	4 (0–8)	9 (6–10)	< 0.001
MNA-SF, median (IQR)	8 (5–10)	9 (7–11)	0.004
Drugs, median (IQR), range	5 (3–7), 0–14	5 (3–7), 0–13	0.3
Co-habitation, n (%)			
Living with family	45 (43.2)	65 (46.4)	0.2
Institute	19 (18.2)	14 (10.0)	
Living alone	40 (38.5)	61 (43.6)	

Mean values of Exton Smith Scale are not available; *p value for Mann–Whitney test or Fisher exact test.

*IQR* interquartile range, *ADL* activities of daily living, *CIRS* cumulative illness rating scale, *IADL* instrumental activities of daily living, *IQR* interquartile range, *MNA-SF* mini nutritional assessment short form, *MPI* multidimensional prognostic index, *SD* standard deviation, *SPMSQ* short portable mental status questionnaire.

In a multivariate logistic model adjusted for potential pre-, intra-, and post-operative confounders including gender, age, social support network, number of drugs, waiting time for surgery, type of anesthesia, and presence of infection or anemia after surgery, high-risk MPI category (MPI-3) at admission was independently associated with higher risk of pre-operative delirium compared to subjects in intermediate risk category (MPI-2) [OR 2.45, confidence interval (CI) 1.21–4.96, *p* = 0.01] (Table 3).

**Table 3:** Multivariate logistic model for the diagnosis of pre-operative delirium.

Factor	Odds ratio	95% CI	*p* Value
**Pre-operative characteristics**			
MPI class			
2	1.0		
3	2.45	1.21–4.96	0.01
Age	1.08	1.03–1.14	0.001
Social support network			
Living with family	1.0		
Institute	1.13	0.45–2.86	0.8
Living alone	0.85	0.43–1.66	0.6
**Intraoperative characteristics**			
Anesthesia			
Spinal	1.0		
Spinal + nerve blocks	0.74	0.40–1.37	0.3
General	1.10	0.17–7.34	0.9
Other	0.19	0.06–0.66	0.009
**Post-operative characteristics**			
Anemia			
No	1.0		
Yes	2.12	1.08–4.16	0.03
Infection			
No	1.0		
Yes	2.03	1.12–3.69	0.02

Odds ratios are also adjusted for gender, waiting time for surgery and number of drugs.

*CI* confidence interval.

## Discussion

In the present study, we demonstrated that just from a standard CGA it is possible to obtain useful information to identify subjects at risk for delirium. Specifically, high-risk MPI category was associated with occurrence of pre-operative delirium among older adults undergoing surgery for hip fracture.

Seniors with hip fracture are very vulnerable subjects at elevated risk of mortality^27^. Indeed, in our cohort, we did not find any patient in MPI-1, the low mortality risk category. Multidimensional impairment before surgery could also identify subjects more prone to develop life-threatening complications as pre-operative delirium. Incidence of pre-operative delirium in our cohort was 43% which is similar to previously reported estimates in older adults with hip fracture^3,9^. In particular subjects with higher MPI score (MPI-3) at admission had 2.4 times higher risk to develop delirium before surgery, independently by other potential confounders.

The mechanisms underlying pre-operative delirium are still unclear. They might be partially different also from those of post-operative delirium, and mainly related to fracture-associated pain and adverse effects of analgesic treatments^3^. A number of risk factors favoring delirium occurrence have been recognized and can be distinguished between predisposing and precipitating factors^28,29^. Here, we found that elderly subjects who experienced pre-operative delirium were significantly older, and already more compromised at admission, having lower cognitive performance, poorer functional status and being more malnourished compared to those patients who did not have delirium. Post-operative complications as infections and anemia were associated with presence of delirium before surgery. Overall post-operative complications could explain also the longer length of in-hospital stay in the delirium group. In a recent meta-analysis, Smith et al. revised 32 studies for a total of 6704 included older adults with hip fracture^30^. They assessed potential pre-, intra-, and post-operative risk factors for delirium. Consistently with our findings, they demonstrated that people with delirium are roughly three years older, more often institutionalized prior the hospital admission, and have lower cognitive scores as assessed by Mini Mental State Examination (MMSE)^30^. Presence of dementia at admission and higher American Society for Anesthesiologists (ASA) score (i.e. grade III and IV) are associated with six- and two-time higher risk of delirium, respectively^30^. Other reports from older adults with hip fracture showed that risk factors for pre-operative delirium are partially different from those of post-operative delirium^9,16^. Specifically, waiting time to surgery, number of comorbidities, use of opioids and benzodiazepines, and fever were associated with pre- but not post-operative delirium^9,16^.

Our study demonstrated that pre-surgical multidimensional assessment using the MPI, a prognostic index based on data available from a standard CGA, was associated with occurrence of delirium independently by age and other potential confounders intervening later during hospitalization (e.g. delay of surgery, type of anesthesia, infections, anemia). Several prediction models have been proposed to identify in-patient older adults at risk for delirium^29,31–35^, but few instruments have been validated specifically for detection of patients more prone to develop pre-operative delirium. In particular, the delirium elderly at risk (DEAR) tool, has been developed to predict incidence of delirium before surgery among older adults with hip fracture^36^. It is a five-item scale assessing cognitive deficits, sensory impairment, functional dependence, substance use, and age (> 80 years old), with a score ranging from 0 (no risk factor) to 5 (all risk factors)^36^. However, the DEAR tool, using a cut-off value of 3, showed good specificity (82%), but quite low sensitivity (63%) in predicting pre-operative delirium^36^. Collectively these data support the concept that multidimensional aggregate information, readily available in clinical practice and easy to obtain, could help physicians to predict occurrence of pre-operative delirium in older patients with hip fracture.

The present study has also some limitations. Firstly, since the study population included selected patients, it is possible that the sample is unrepresentative of older population hospitalized with hip fracture. Secondly, the retrospective design did not allow to systematically collect further information for example about: type of fracture, mechanism of injury, ASA score, delirium motor subtypes (hyperactive, hypoactive, mixed), analgesic and sedative treatments, or other potential post-operative complications. Finally, the study population was relatively small, and the patients were recruited from a single hospital. Therefore, larger prospective multicenter studies are needed to confirm and validate these findings.

In conclusion, the care of hospitalized older adults with hip fracture, who are at risk for delirium, requires a collaborative multidisciplinary effort involving geriatricians, orthopedic surgeons, anesthesiologists, and nurses. The CGA-based MPI, collected at hospital admission, might be a sensitive tool to early identify subjects at risk to develop pre-operative delirium and thus could represent a crucial step toward individualized decision making.

## Data Availability

The dataset is available from the corresponding author on reasonable request.

## Code availability

All analyses were conducted using STATA software, and the code is available on request.
